# Surface Modification of PP and PBT Nonwoven Membranes for Enhanced Efficiency in Photocatalytic MB Dye Removal and Antibacterial Activity

**DOI:** 10.3390/polym15163378

**Published:** 2023-08-11

**Authors:** Shahad M. Aldebasi, Haja Tar, Abrar S. Alnafisah, Hanène Salmi-Mani, Noura Kouki, Fahad M. Alminderej, Jacques Lalevée

**Affiliations:** 1 Department of Chemistry, College of Science, Qassim University, Buraidah 51452, Saudi Arabia; 411207279@qu.edu.sa (S.M.A.); f.alminderej@qu.edu.sa (F.M.A.); 2 Institut de Chimie Moléculaire et des Matériaux d’Orsay, Université Paris-Saclay, CNRS, 91405 Orsay Cedex, France; hanene.salmi@u-psud.fr; 3 CNRS, IS2M UMR 7361, Université de Haute-Alsace, F-68100 Mulhouse, France; jacques.lalevee@uha.fr

**Keywords:** methylene blue (MB), polypropylene (PP), poly(butylene terephthalate) (PBT), nanoparticles, surface modification

## Abstract

In this study, we developed highly efficient nonwoven membranes by modifying the surface of polypropylene (PP) and poly(butylene terephthalate) (PBT) through photo-grafting polymerization. The nonwoven membrane surfaces of PP and PBT were grafted with poly(ethylene glycol) diacrylate (PEGDA) in the presence of benzophenone (BP) and metal salt. We immobilized tertiary amine groups as BP synergists on commercial nonwoven membranes to improve PP and PBT surfaces. In situ Ag, Au, and Au/Ag nanoparticle formation enhances the nonwoven membrane surface. SEM, FTIR, and EDX were used to analyze the surface. We evaluated modified nonwoven membranes for photocatalytic activity by degrading methylene blue (MB) under LED and sunlight. Additionally, we also tested modified membranes for antibacterial activity against *E. coli*. The results indicated that the modified membranes exhibited superior efficiency in removing MB from water. The PBT showed the highest efficiency in dye removal, and bimetallic nanoparticles were more effective than monometallic. Modified membranes exposed to sunlight had higher efficiency than those exposed to LED light, with the PBT/Au/Ag membrane showing the highest dye removal at 97% within 90 min. The modified membranes showed reuse potential, with dye removal efficiency decreasing from 97% in the first cycle to 85% in the fifth cycle.

## 1. Introduction

Significant progress has been made in the research and development of polymer composites and photocatalytic degradation for water treatment [1,2,3]. These advancements aim to improve environmental sustainability and address the challenges posed by water pollution caused by organic dyes such as MB [4,5]. Researchers have explored various advanced oxidation processes (AOPs) [6], including photocatalytic degradation [7], ozonation [8], and UV/H_2_O_2_ oxidation [9], to remove MB and other textile dyes from wastewater. Several researchers have modified nonwoven fabric membranes for photocatalytic degradation of MB [10,11]. The incorporation of metals, nonmetals, and semiconductor heterojunctions into polymer-based photocatalysts shows promise for enhancing their performance in water treatment applications [12,13].

Nonwoven fabric membranes, which are web structures consisting of randomly bonded fibers and filaments, are widely used in various applications ranging from medicine to electronics [14]. These nonwoven fabrics possess several distinctive features, including an engineered interconnected porous structure, a relatively large surface-to-volume ratio, porosity, controllable pore size, fiber shape, fiber diameter, and a low production cost. However, they also have limitations such as low specific surface areas, chemical inertness, and hydrophobic nature [15,16]. To overcome these limitations, surface modification techniques can be employed to introduce chemical functional groups and enhance the hydrophilicity of nonwoven fabric membranes. One effective method is surface-initiated polymerization, which involves grafting a thin polymer layer onto the membrane surface, altering its surface properties [17,18]. In industries, UV irradiation and plasma treatments are commonly used to enhance the hydrophilicity of hydrophobic surfaces such as poly(butylene terephthalate) (PBT) and polypropylene (PP). UV irradiation, in particular, is successful in introducing functional groups onto polymeric surfaces without damaging the bulk material [19,20]. By applying these surface modification techniques, nonwoven fabric membranes can be tailored to exhibit improved properties such as enhanced hydrophilicity and the introduction of functional groups [17]. These modifications contribute to expanding the potential applications of nonwoven fabrics in fields like water treatment and biomedical applications, where hydrophilicity and specific surface properties are crucial [19,20].

Surface-initiated polymerization techniques are commonly used for easily modifying surfaces on various materials [21]. A two-step photo-grafting technique has been employed by multiple research groups using surfaces capable of providing hydrogen for abstraction, such as polyethylene terephthalate and polylactic acid [22,23]. To initiate free radical polymerization through hydrogen abstraction under UV light, benzophenone (BP) is widely utilized as a type II photoinitiator in photo-induced surface grafting [24]. Castell et al. conducted experiments to alter the surface characteristics, particularly wettability and adhesion, of chemically inert polypropylene films using BP and its derivatives without subsequent polymerization. They observed an increase in surface energy from 26 (in pure PP) to 36 mN/m, depending on the structure of the BP derivative used. Subsequently, they pursued enhanced surface wettability and adhesion through the process of photografting, grafting monomers like methacrylic acid (MAA), pentaerythritol triacrylate, and 2-hydroxyethyl acrylate onto benzophenone-derivatized polypropylene (PP) surfaces [25,26]. Zheng et al. demonstrated the formation of highly conformal and uniform polyGMA grafts on PP nonwoven fabrics by employing a UV pretreatment step followed by UV grafting with BP as the initiator [27]. Liu et al. developed a potential disposable affinity membrane for pathogen removal from biological systems by grafting poly glycidyl methacrylate (polyGMA) onto the fiber surface of a nonwoven poly(butylene terephthalate) (PBT) using photo-induced graft polymerization with BP [28]. In a recent study, polyacrylic acid (PAA) was grafted onto a polyamide (PA) surface through UV-initiated graft polymerization in the presence of BP as a photoinitiator to enhance the pervaporative dehydration of aqueous alcohol solutions [29].

Numerous researchers have utilized various vinyl monomers, including acrylic acid and poly(ethylene glycol) methacrylates, to modify membrane surfaces [30,31]. Among these hydrophilic monomers, poly(ethylene glycol) (PEG) and its derivatives have received considerable attention due to their excellent antifouling properties. PEG is a water-soluble polymer with uncharged characteristics, strong hydrophilicity, flexible long chains, a large exclusion volume, and unique coordination with water molecules. By grafting PEGDA (poly(ethylene glycol) diacrylate) onto fiber surfaces, the hydrophilicity of the membranes can be significantly enhanced [32].

In recent years, there has been a growing interest in exploring the photocatalytic degradation properties of metal/polymer nanocomposites. Studies have demonstrated that incorporating noble metal nanoparticles into nanocomposites greatly enhances their catalytic activity and antibacterial activity [12,13,33]. Noble metal nanoparticles such as gold (Au) or silver (Ag) exhibit distinct optical properties, primarily absorbing visible and UV light due to phenomena such as surface plasmon resonance (SPR) and interband electron transitions [34,35,36,37,38,39]. These properties make metal nanoparticles highly effective in photocatalytic materials used for various processes, including organic pollutant mineralization and dye removal [12,34,40,41]. Recent research has also focused on investigating the photocatalytic degradation activities of metal nanoparticles embedded in polymer matrices, and it has been found that the addition of gold and silver nanoparticles enhances the catalytic activity of the polymer matrix [12,13]. Bimetallic composites offer significant advantages over individual metallic nanocomposites, displaying superior physical and chemical properties such as high activity, functionality, and stability due to synergistic effects [42]. Furthermore, incorporating silver nanoparticles onto membrane surfaces has been shown to improve hydrophilicity and enhance antibacterial properties, according to recent studies [43,44,45].

This study focuses on the modification of two types of nonwoven membranes based on PP and PBT through photo-polymerization in a polar organic solution. The modification process involves two steps: aminolysis and photo-grafting polymerization. In the first step, a tertiary amine co-initiator is employed to enhance reactivity, with the amino-substituted alkyl radical considered as the primary initiating radical [46,47]. Previous research has indicated that amine-based membrane modifications can improve their hydrophilic properties [48,49]. Following the aminolysis, the membranes undergo photo-grafting polymerization using poly(ethylene glycol) diacrylate in the presence of benzophenone and a metal salt. Gold, silver, and bimetallic nanoparticles are utilized to enhance the surface properties of the membranes. The main objective of this study is to compare the efficiency of the two modified nonwoven membrane types in removing methylene blue from water under LED and sunlight irradiation conditions. Additionally, the antibacterial activity of the modified membranes is also being investigated. The LED irradiation used in the study has a wavelength of 419 nm and an intensity of 250 (mW/cm^2^).

## 2. Materials and Methods

### 2.1. Materials

Custom polypropylene (PP) nonwovens (0.30–0.35 mm of thickness) from MacoPharma (Mouvaux, France) were used in this study. They were obtained by melt-blown technology. It should be noted that an O_2_-plasma treatment was performed by the supplier, MacoPharma, giving a hydrophilic behavior to the nonwoven PP material, which is initially hydrophobic. Custom poly(butylene terephthalate) (PBT) nonwovens (thickness 0.26–0.36 mm; density) was also purchased from MacoPharma. This material was obtained by melt-blown technology.

**Scheme 1:**
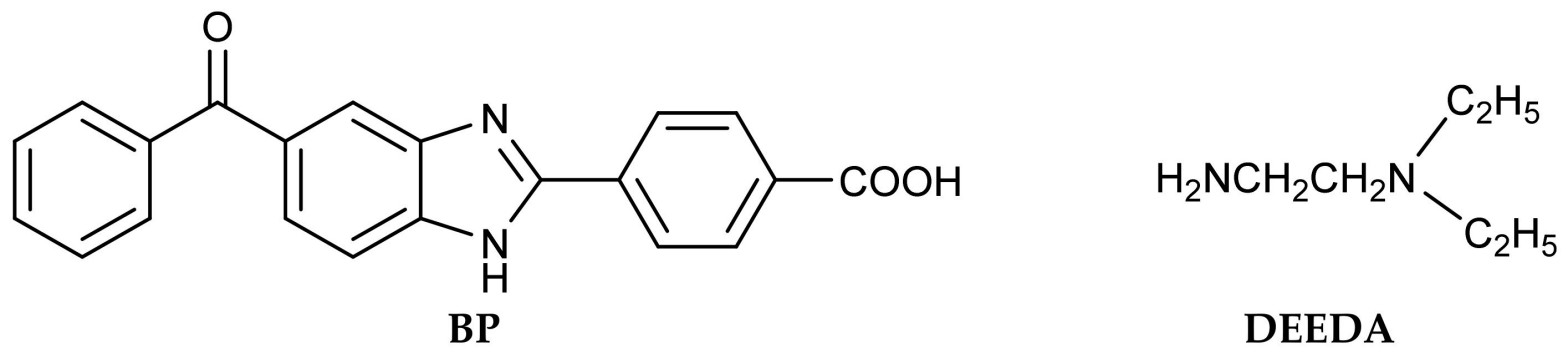
The chemical structure of the benzophenone (BP) and amine used in this study.

The structure of the photoinitiator (benzophenone derivative BP) is shown in Scheme 1, and the synthetic process for the benzophenone derivative can be found in our previous study [50]. Silver nitrate (AgNO_3_, 99.99%), gold (III) chloride hydrate (HAuCl_4_, 99.99%), methylene blue (MB), N,N-diethylethylenediamine (DEEDA, for synthesis), orange II sodium salt (Dye content ≥ 85%), poly(ethylene glycol) diacrylate (PEGDA, Mn = 700, for synthesis), anhydrous citric acid, acetic anhydride, sodium hydroxide, hydrochloride, ethyl acetate, and methanol were purchased from Sigma Aldrich (Burlington, MA, USA).

### 2.2. Surface Modification of Nonwoven Membrane

#### 2.2.1. Synergist Immobilization by Aminolysis

Three milliliters of DEEDA were added to a sealed glass container containing the nonwoven membrane sample with a diameter of 1 cm^2^. After 10 min at room temperature to achieve thermal equilibrium, the system reacted in a thermostat at a steady temperature for a specified period. Subsequently, the membranes were extracted from the vessel and washed with methanol three times (each for 10 min), and dried at 45 °C overnight.

#### 2.2.2. Determination of Tertiary Amino Groups

A solution referred to as the “D-solution” was prepared using a mixture of 150 mM citric acid in a combination of acetic anhydride and ethyl acetate (*v*/*v* = 1:1). The membranes, whether original or aminolyzed, were introduced into the D-solution. The reaction took place at a temperature of approximately 50 °C, and the resulting color change of the membranes was observed. This allowed for qualitative evaluations to be made regarding the efficiency of immobilizing the synergist [47]. 

A quantitative staining method with the anionic dye AO was used to determine the amount of tertiary amino groups in the membrane samples that had been aminolyzed [47,51]. The membrane samples were stored at room temperature in a 0.5 mM AO in water (pH 3, HCl) solution overnight with shaking. The ionically bound dye was eluted by first washing the samples three times with water (pH 3), and then immersing them in 10 mL of water (pH 12, NaOH). The absorption of the solution was measured after 15 min of shaking (Figure S1). When AO was dissolved in a pH 12 aqueous solution, a calibration curve was formed (Figure S2).

#### 2.2.3. Photo-Grafting Polymerization In Situ Nanoparticles

The aminolyzed nonwoven membrane was modified via photopolymerization by mixing poly(ethylene glycol) diacrylate (PEGDA) with various ratios of metal salt in the presence of the BP in methanol according to the mass listed in Table 1. Aminolyzed membranes were immersed in monomer formulations on glass plates and exposed to a LED with a wavelength of 419 nm and an intensity of 250 (mW/cm^2^) for a certain period of time. Subsequently, the samples were promptly extracted and rinsed three times with water for a duration of 10 min each to remove any unreacted monomer, photoinitiator, or homopolymer. Afterward, the membranes were exposed to a drying process at a temperature of 45 °C for the duration of one night.

The degree of grafting DG (%) was determined gravimetrically from the weight of each sample before and after modification though following equation [52], Where *W*_0_ and *W*_g_ are the weights of membrane before and after grafting, respectively:

(1) $$Degree\;of\;grafting\left(\%\right)=\frac{W_{g}–W_{0}}{W_{0}}\times 100$$

### 2.3. Membrane Surface Characterization

FTIR is measured using Jasco 6400 in the range 4000–400 cm^−1^ with resolution 4 cm^−1^. The morphology of the nonwoven membrane surface was examined by a field emission scanning electron microscope (FESEM) (JEOL JSM 6490-A) at different resolutions.

### 2.4. Photocatalytic Activity 

The photocatalytic activity of the modified nonwoven membrane was evaluated following the photodegradation of methylene blue (MB) in aqueous solutions under 419 nm irradiation with an intensity of 250 (mW/cm^2^). Then 5 mL of an aqueous solution of MB (5 ppm) containing the grafted nonwoven membrane (1 cm^2^) was irradiated for 90 min. The progress of the photodegradation was followed by a Shimadzo UV-1800 spectrophotometer (Shimadzo, Duisburg, Germany) at different times. The same procedure was carried out on the identical sample under sunlight as a light source. It is important to acknowledge that the experiments were conducted outdoors in the Kingdom of Saudi Arabia, exposed to direct sunlight at a temperature of 39 °C.

The reusability of the modified membrane was tested by using the same sample in five successive cycles for MB photodegradation (5 ppm). The membrane was washed with water, kept dry in the dark at room temperature, and used again in the photodegradation of MB. The progress of the photodegradation was followed by UV-vis spectrophotometry, as previously described.

### 2.5. Antibacterial Test

The PP/Au/Ag and PBT/Au/Ag membranes underwent filtration using a vacuum flask filtration setup with 50 mL of *E. coli* suspension. The sterilization process involved exposing all glasses to a temperature of 121 °C for 20 min. The suspension was filtered through an untreated nonwoven membrane, and the bacterial growth was evaluated on agar plates. A suspension was introduced into both the treated and untreated membranes and subsequently incubated for 96 h to observe the growth of bacteria.

The treated nonwoven membrane was also tested against *E. coli* by a suitable diffusion method to test the antibacterial activity of the treated membrane. The bacterial suspension was cultured in a nutrient broth medium under optimal conditions of 37 °C for 24 h. Drops of bacterial suspension (100 µL) were added to the surface of the agar and subsequently spread using a sterile glass spreader. A 7 mm diameter well was made on a nutrient agar plate with the help of a gel puncture. Then 1 cm of nonwoven membrane was added to the well, and then the plates were incubated in an incubator at 37 °C for 24 h. The standard error was calculated using three replicated experiments.

## 3. Results and Discussion

### 3.1. Surface Characterization of the Grafting Nonwoven Membrane 

#### 3.1.1. Modification of PP and PBT Membranes Surface by Photoinitiated Process 

**Scheme 2:**
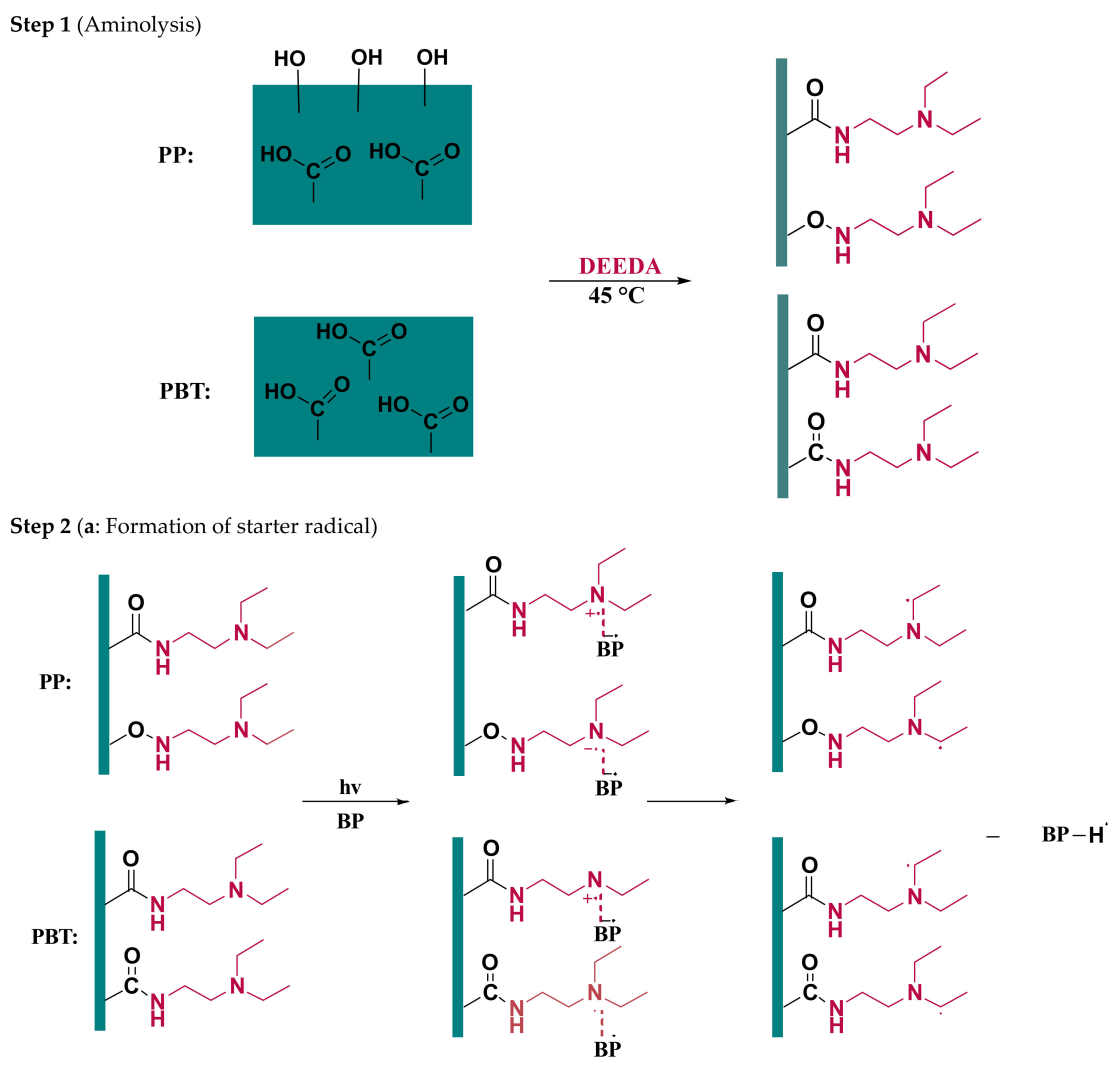

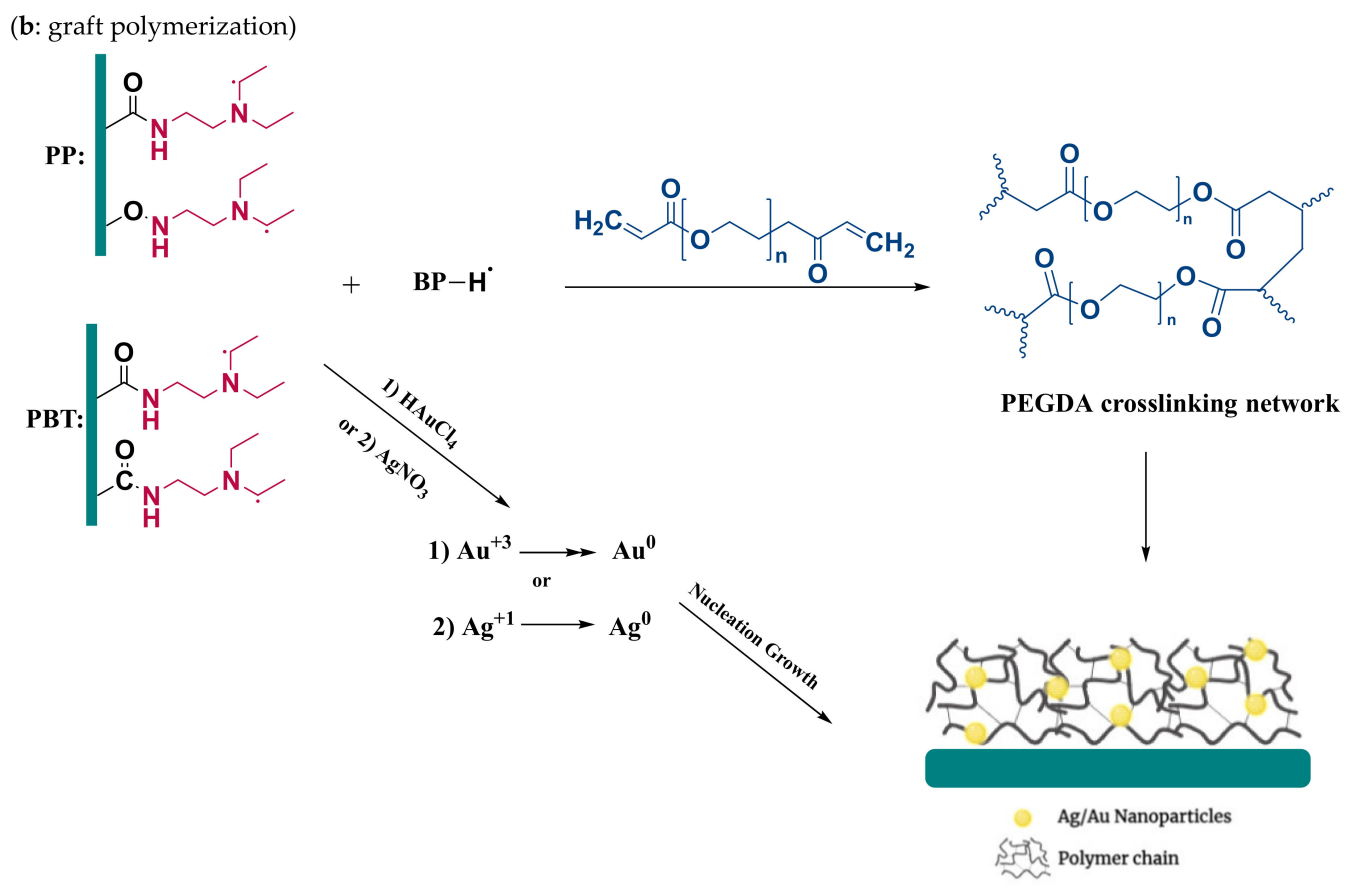
Proposed mechanism for the photo-grafting PP and PBT.

The nonwoven membrane surface underwent functionalization with tertiary amino groups by utilizing *N*,*N*-diethylethylenediamine (DEEDA) through an aminolysis treatment. In this process, a nucleophilic attack of DEEDA primary amines took place on the carbonyl carbon associated with plasma-activated PP and PBT materials, resulting in the creation of an amide function on the membrane surface (Scheme 2, step 1).

Upon completion of the aminolysis process, tertiary amines became covalently grafted onto the surface of PP and PBT membranes. These aminolyzed membranes contained tertiary amines that act as efficient co-initiators for the activation of the photopolymerization process in the presence of a photoinitiator such as BP [47].

When the surface of the membrane exposed to LED light in the presence of benzophenone derivative (BP), the excited state of BP interacts with the ground state of the tertiary amino groups grafted onto the surface, following a photoinduced electron transfer mechanism. This leads to a hydrogen abstraction reaction, resulting in the formation of a radical from the benzophenone (ketyl radical) and an aminoalkyl radical derived from the tertiary amine functions (Scheme 2, step 2a). 

In the presence of poly(ethylene glycol) diacrylate (PEGDA) as a monomer and the metal salt (AgNO_3_ or HAuCl_4_), the generated amino ketyl radicals efficiently activate the photopolymerization process and at the same time reduce the metal (Au^+3^ to Au^0^ or Ag^+1^ to Ag^0^) leading to the formation of polymer onto the membrane in situ with gold or silver nanoparticles (Scheme 2, step 2b).

#### 3.1.2. Tertiary Amino Group Determination on the Surface of PBT and PP

For the identification of tertiary amine, the method D-solution was used (see Section 2.2.2). The aminolyzed nonwoven membrane exhibited a wine-red color (Figure S3), whereas the control samples did not manifest any discernible coloration. Hence, the aminolysis seemed to proceed successfully under the selected conditions [47]. 

A quantitative value of tertiary amino groups grafted on PP and PBT surfaces was determined via the colorimetric method using the anionic dye orange II (Figures S1 and S2). The concentration of amine grafted on the surface of PP and PBT were calculated to be around 7.9 and 14 nmol/cm^2^, respectively. This later, indicates that more amine groups have been grafted onto the PBT compared to the PP membrane. The PBT membrane contains ester groups, which can provide more sites for grafting reactions. The presence of these functional groups may facilitate amine grafting, potentially leading to a higher concentration of amine on the PBT compared to the PP membrane.

#### 3.1.3. FTIR Analysis 

**Figure 1:**
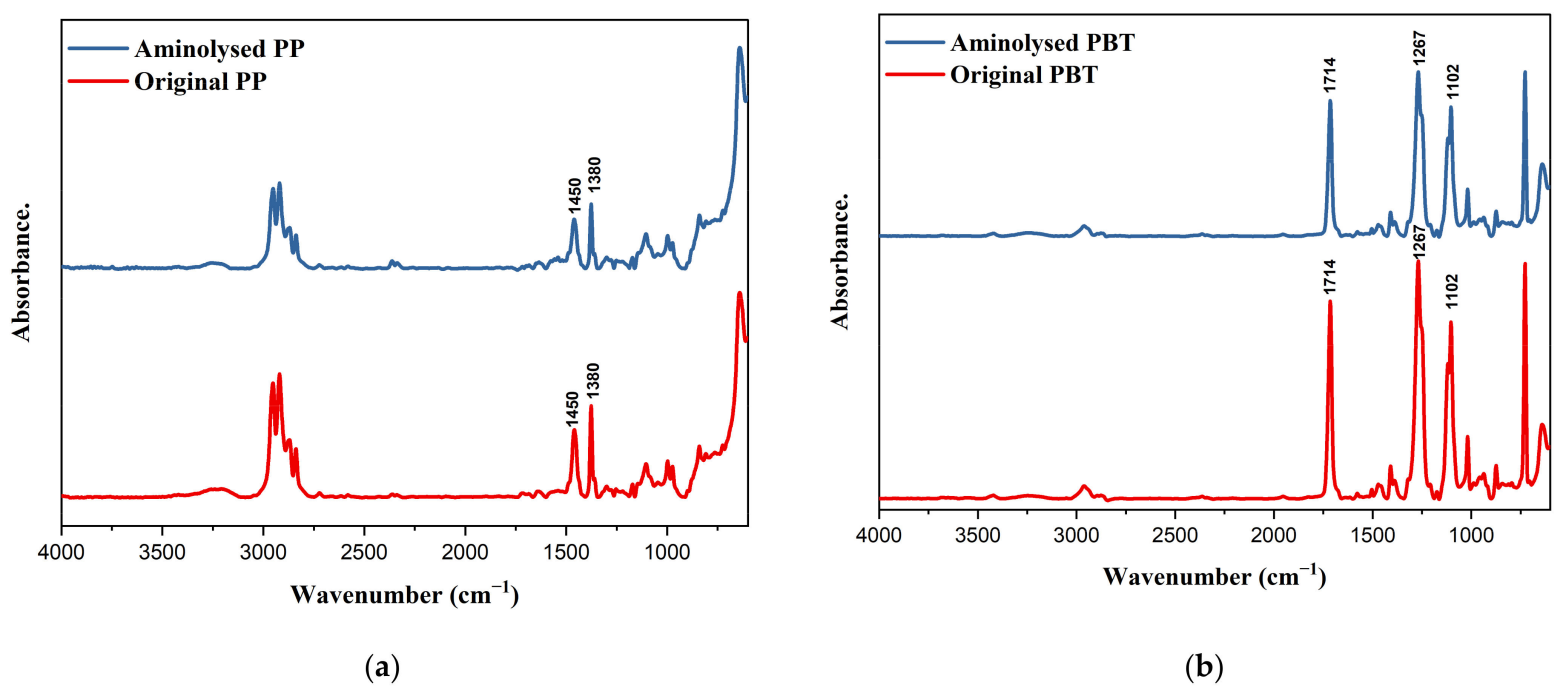
FT-IR spectra for: (**a**) original and aminolyzed PP nonwoven membranes; (**b**) original and aminolyzed PBT nonwoven membranes.

FT-IR spectra of PP and PBT samples that have undergone aminolysis are presented in Figure 1, with the spectra of their respective blank samples.

The presence of characteristic peaks at 1380 and 1450 cm^−1^ in Figure 1a are an indication of PP [27]. In addition, carbonyl band was observed at 1550–1900 cm^−1^ [53]. Figure 1b peak showed at 1714 cm^−1^ corresponds to carbonyl group (C=O), peaks at 1267 cm^−1^ and 1102 cm^−1^ correspond to ester group from PBT [28]. From FT-IR spectra of aminolyzed membranes, no amide absorption peaks could be detected after the reaction. 

**Figure 2:**
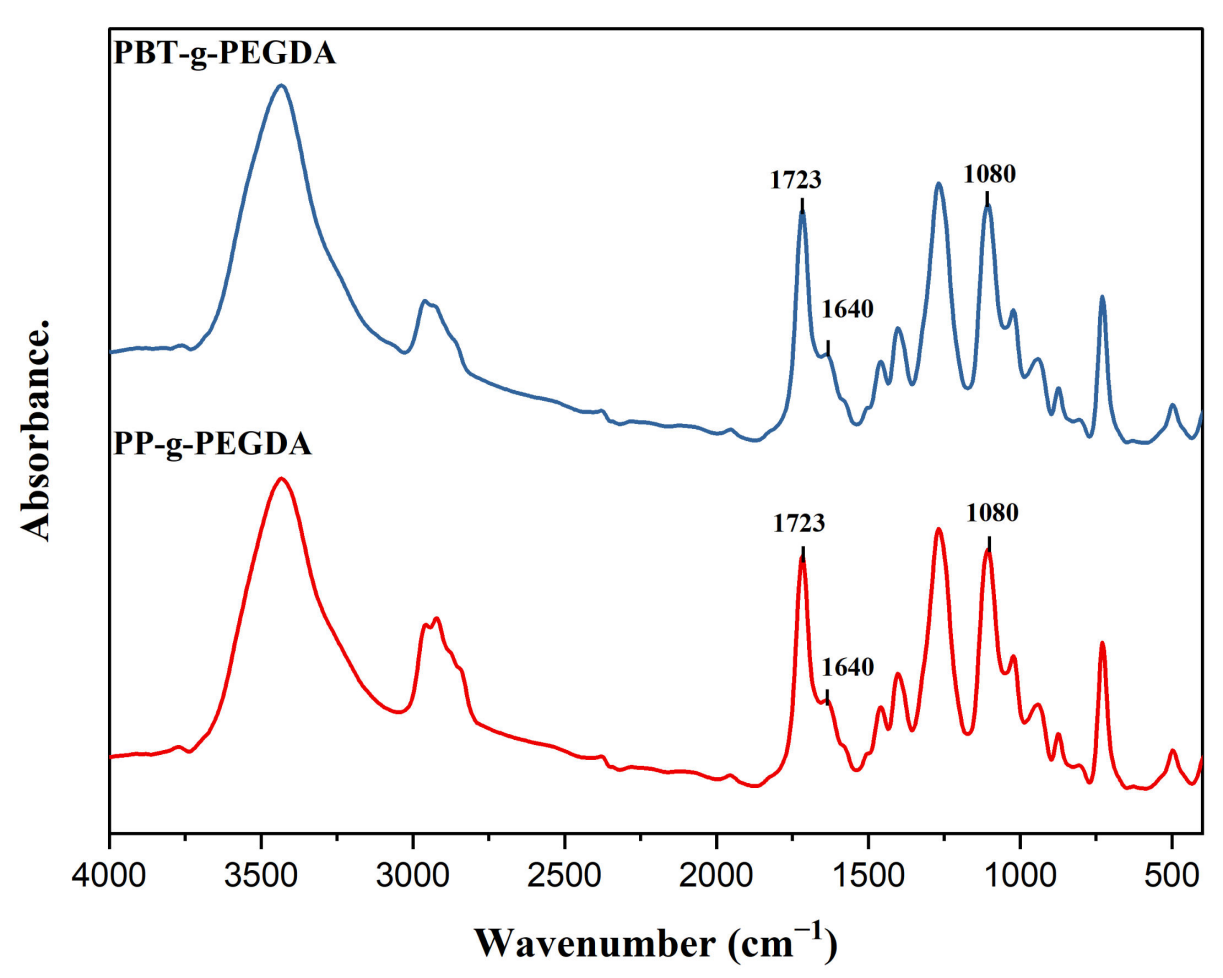
FT-IR spectra for grafted PP and PBT nonwoven membranes after photopolymerization with PEGDA.

Figure 2 depicts the FT-IR spectra of the grafted PP and PBT nonwoven membranes with PEGDA. Both grafted PP and PBT nonwoven membranes exhibited an absorption band at 1080 cm^−1^ which indicates the presence of the ether moiety of polyethylene glycol (PEGDA), which is frequently employed for the identification of PEGDA on membrane surfaces [54,55,56]. As a point of reference, the peak intensity at 1723 cm^−1^ corresponded to the stretching vibrations of the carbonyl group (C=O) in PEGDA [57]. Additionally, the disappearance of the double band at 1640 cm^−1^ contributed to the consumption of (-C=C-) bonds during photopolymerization [58]. The degree of grafting of polymer on the surface was obtained in the range of 35–40%. Figure S4 depicts images of samples after the grafting.

#### 3.1.4. EDX and SEM Characterization 

**Figure 3:**
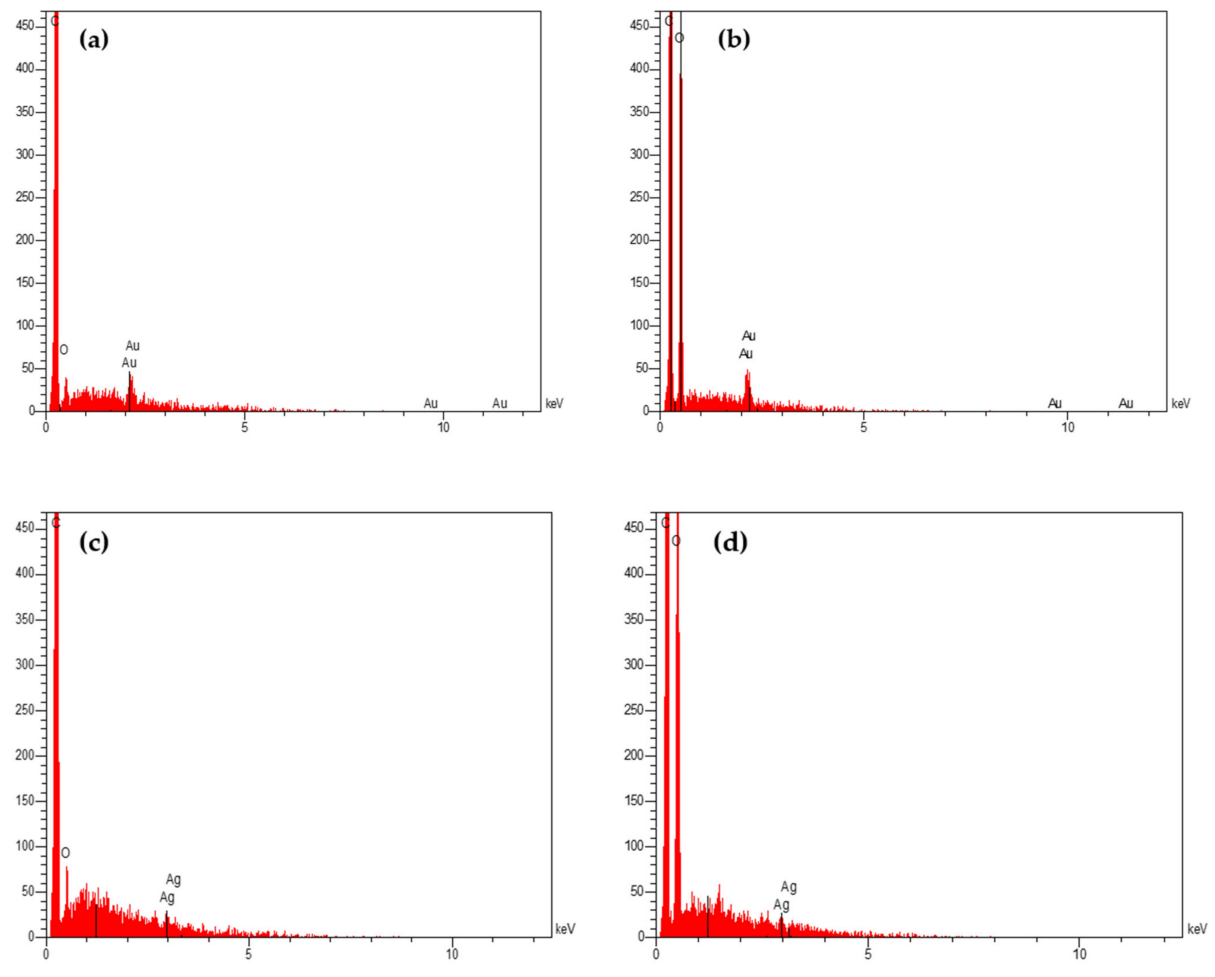
EDX of PP and PBT nonwoven membranes after grafting: (**a**) PP/Au, (**b**) PBT/Au, (**c**) PP/Ag, and (**d**) PBT/Ag.

Figure 3 depicts the EDX spectrum of the nonwoven membrane grafting in fracture, used to confirm the elemental composition of the photo-crosslinked samples. The presence of the Au element is evidenced by the peaks observed at approximately 2.2 keV, as shown in Figure 3a,b. Furthermore, the appearance of the 3.0 keV peaks depicted in Figure 3c,d corresponds to the Ag element, in addition to the peaks attributed to the constituent elements of the organic matrix (C, O) [59]. The SEM analysis confirmed the nanoparticles with a size range from 30 to 120 nm for monometallic NPs and 60 to 200 nm for bimetallic NPs (Figure S5). The sizes of bimetallic NPs were found to be larger compared to monometallic NPs. The same results were confirmed by Melinte and co-workers [59]. Bimetallic NPs may exhibit different size distributions and morphologies compared to their monometallic. The presence of two different metals in bimetallic (Au-Ag) NPs can lead to unique interactions and alloying effects that influence their final size and shape.

**Figure 4:**
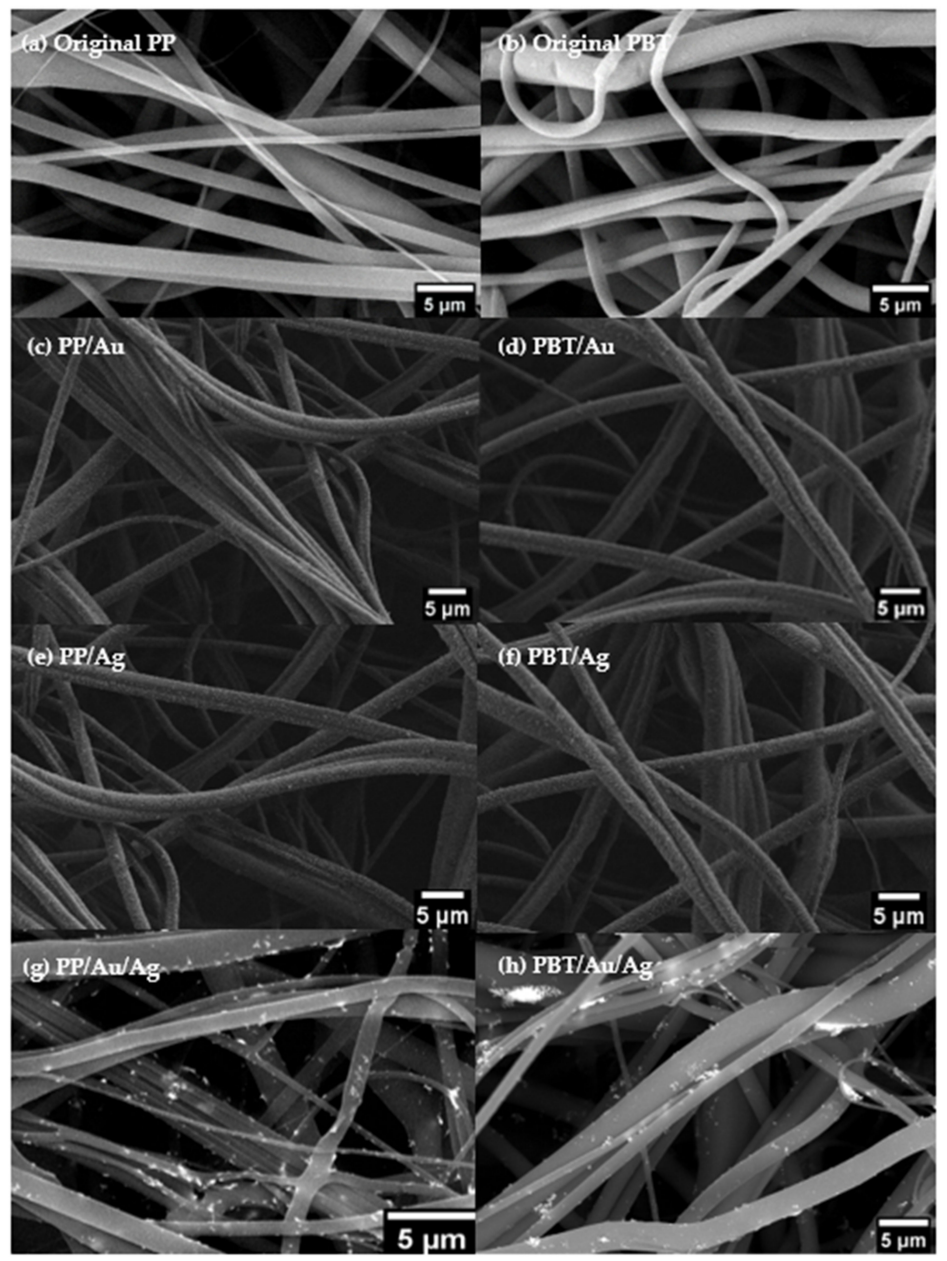
SEM images of the surface of (**a**) original PP, (**b**) original PBT, (**c**) PP/Au, (**d**) PBT/Au, (**e**) PP/Ag, (**f**) PBT/Ag, (**g**) PP/Au/Ag, (**h**) PBT/Au/Ag.

The SEM images were also utilized to investigate the surface morphologies of the nonwoven membranes both before and after grafting. This later, depicted in Figure 4a,b illustrate the surface morphology of blank PP and PBT membranes with a random network of fibers. The original nonwoven membrane exhibited a relatively smooth surface morphology of fibers. The grafted layer is responsible for the discernible surface roughness observed in the depicted nonwoven membrane in Figure 4c–h. This feature is absent in the unmodified counterpart. A similar distribution of white-colored grains has been noted on the grafted samples, which could be attributed to the presence of a grafting layer on the nonwoven fibers, as depicted in Figure 4c–h. 

### 3.2. Photocatalytic Activity Evaluation 

Methylene blue (MB) is a cationic dye that is extensively used as a model dye to investigate practical usage in photocatalytic applications [60]. The photodegradation of MB in the presence of grafted membranes as catalysts under different light sources was investigated. The change in optical absorption spectra of MB by various nonwoven membrane samples exposed to a LED with a wavelength of 419 nm and an intensity of 250 (mW/cm^2^) or sunlight irradiation was studied.

The photocatalytic degradation efficiencies of samples were determined by using the equation given below [61]:

(2) $$\mathrm{Percentage}\;\mathrm{of}\;\mathrm{degradation}\left(\%\right)=\frac{C_{0}-C_{t}}{C_{0}}\times 100$$

where $C_{0}$ and $C_{t}$ are the concentration of MB before irradiation and the MB concentration after irradiation at a given time, respectively.

#### 3.2.1. Photocatalytic Activity under LED Light 

**Figure 5:**
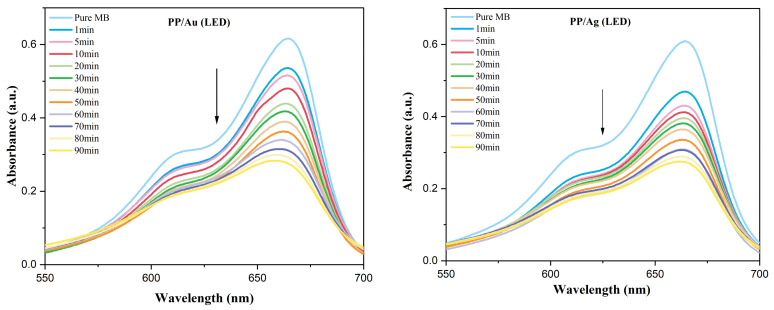

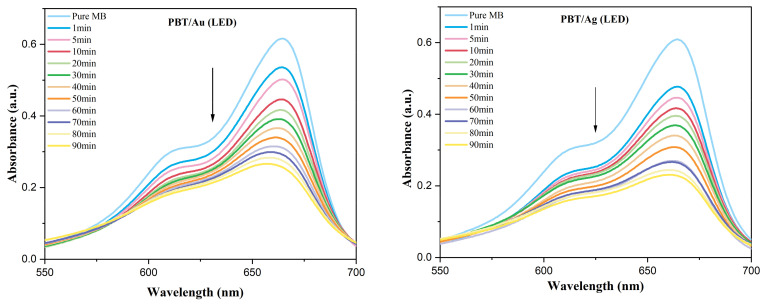
UV-Vis absorption spectrum of the photodegradation of PP/Au, PP/Ag, PBT/Au, and PBT/Ag. Membrane samples (1 cm^2^ in 5 mL) depending on the time under LED with a wavelength of 419 nm irradiation.

The MB dye absorbs light most strongly at around 663 nm in the visible region [62]. When exposed to LED light with a wavelength of 419 nm and an intensity of 250 mW/cm^2^, the peak absorption at 663 nm gradually decreases with increasing irradiation time. In all photodegradation experiments, there is a slight shift of the absorption bands towards lower wavelengths over time. This shift is attributed to the N-demethylation of MB molecules during LED-induced photodegradation [63]. Figure 5 shows the UV-visible absorption spectra of MB solutions at different time intervals using irradiation at 419 nm for various samples. The degradation percentage of the samples within a total reaction time of 90 min is presented in Table 2. Among the PP nonwoven membrane samples, the best degradation efficiency was observed with PP/Ag (55.1%). Compared to PP/Au, which is the same sample modified with gold instead of silver, gold exhibited lower efficiency with a degradation ratio of 53.8%. The PBT nonwoven membrane outperformed the PP nonwoven membrane in all samples, with degradation ratios of 62% and 56.6% for PBT/Ag and PBT/Au, respectively. The increased reactivity of the PBT membrane can be explained by the higher percentage of grafting achieved compared to the PP membrane. Additionally, it is worth noting that the presence of a carboxylic acid group in the initiator enhances the negative charge on the surface. Moreover, some studies have shown that introducing the carboxylic group on the surface of the fibers improves the dye’s ability to absorb light [1,64].

#### 3.2.2. Photocatalytic Activity under Sunlight 

As the majority of the solar radiation intensity reaching the earth surface is in the visible range (400–600 nm), visible-light active systems have become a priority for developing the future generation of photocatalytic materials [65,66]. In order to use sunlight energy effectively, the design and development of photocatalytic systems capable of operating under visible or solar light irradiation have been desired for the applications of photocatalytic systems. especially for environmental concerns [67].

##### 3.2.2.1. Surface Modified In-Situ Monometallic Nanoparticles 

**Figure 6:**
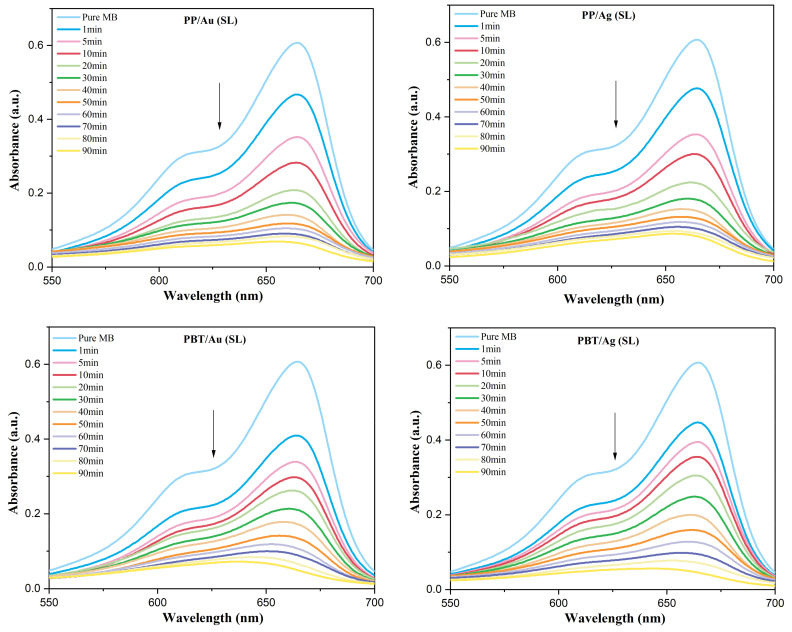
UV-Vis absorption spectrum of the photodegradation of the; PP/Au, PP/Ag, PBT/Au, and PBT/Ag. Membranes samples (1 cm^2^ in 5 mL) depending on the time under sunlight.

The photocatalytic activity of the modified nonwoven membrane was examined under direct sunlight irradiation, using MB as the target dye, as depicted in Figure 6. PBT samples exhibited superior photocatalytic removal efficiency compared to PP under sunlight conditions, with PBT/Ag (91%) demonstrating the highest degradation capacity, followed by PBT/Au (89%), PP/Au (88%), and PP/Ag (85%) (see Table 3). Notably, the maximum degradation of MB occurred with PBT/Ag (91%) during 90 min of irradiation. Interestingly, the degradation rate of the dye under sunlight was faster than under LED irradiation (at 419 nm wavelength), as sunlight has a higher intensity.

##### 3.2.2.2. Surface Modified In-Situ Bimetallic Nanoparticles 

**Figure 7:**
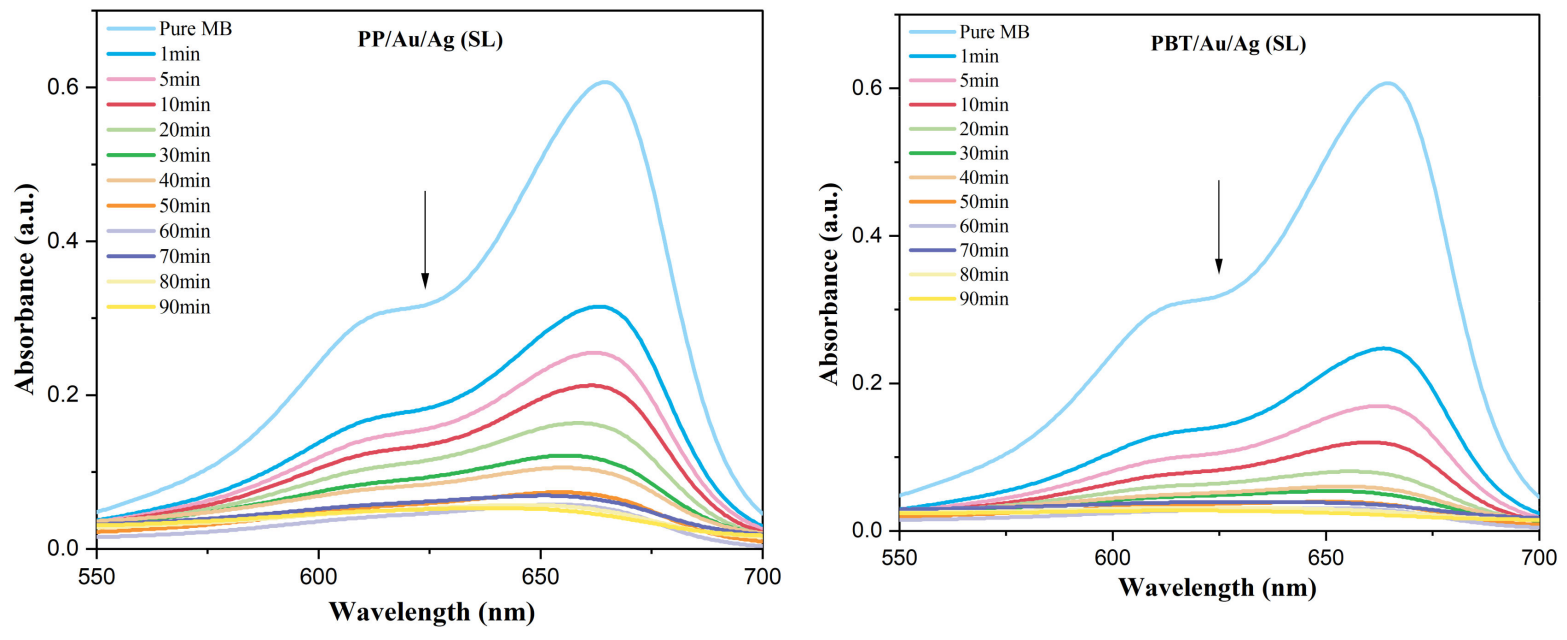
UV-Vis absorption spectrum of the photodegradation of PP/Au/Ag and PBT/Au/Ag. Membranes samples (1 cm^2^ in 5 mL) depending on the time under sunlight irradiation.

Bimetallic nanoparticles are also seen to have increasing importance in addition to metallic NPs, both scientifically and technologically [68]. The use of photocatalysts containing bimetallic nanoparticles immobilized on the nonwoven membrane surface provides many advantages. Both PP and PBT were modified with bimetallic nanoparticles in situ the polymer matrix to study the photocatalytic activity under sunlight (see Table 4). Figure 7 shows that the PBT/Au/Ag exhibited the highest photocatalytic abilities; in particular, the MB almost completely decomposed within 90 min with a degradation efficiency of 97%. The PP/Au/Ag also displayed excellent photocatalytic performance, with a degradation efficiency of 92% within the same time. In general, samples treated with bimetallic nanoparticles removed dye better than monometallic ones. The addition of Au/Ag bimetallic nanoparticle structures provides unique physical and optical properties that are inaccessible to monometallic systems [69]. Due to their decrease in size and increase in surface area, they can be used as catalysts. In the literature, it was found that Au/Ag bimetallic NPs containing hybrid materials showed much higher photocatalytic activity on photocatalytic degradation of organic dyes such as methylene blue when compared to monometallic Au and Ag NPs [34,59,70,71,72,73,74].

### 3.3. Kinetics Study 

The degradation kinetics of MB were examined through the utilization of the pseudo first order model, as presented in the following equation:

(3) $$ln\frac{C_{0}}{C_{t}}=k$$

where $C_{0}$ and $C_{t}$ refer to the concentrations of the dye at time 0 and time *t*, respectively, while k represents the first order rate constant measured in units of (${min}^{-1}$). 

A linear relationship was observed when plotting ln($C_{0}$/$C_{t}$) vs. time. The obtained straight line indicates that the degradation of methylene blue dye through photocatalysis follows first-order kinetics. This is supported by the high correlation constant ($R^{2}$ > 0.95) for the fitted line. The prepared samples exhibited an excellent correlation with regard to first-order reaction kinetics, as depicted in Figure S6. The rate constants (k) for degradation were determined by calculating the slope of the linear regression line based on the first-order kinetic model. It is observed that the rate constant of the degradation reaction was relatively higher under sunlight compared to LED, resulting in the faster degradation of MB under sunlight (Table 5). 

### 3.4. Mechanism for the Degradation of MB 

The crucial involvement of the hydroxyl radical in the degradation process is well known owing to its potent oxidizing ability, which enables it to oxidize a diverse range of organic compounds containing double bonds. The photocatalytic efficacy is contingent upon the production of hydroxyl radicals by the photocatalyst [75].

Photocatalytic reactions on the catalyst surface involve various processes such as absorption of light, generation of charge carriers, transport of electron and hole pairs ($e_{CB}^{-}$/$h_{VB}^{+}$), and surface oxidation-reduction processes [72]. Both the molecules of the catalyst and the dye exhibit efficient absorption of ultraviolet radiation. Upon exposure to light, Au/Ag nanoparticles undergo photon absorption and generate a pair of electrons ($e_{CB}^{-}$) and holes ($h_{VB}^{+}$) as a result of their significant surface plasmon resonance (SPR) effects. The photocatalyst surface facilitates the reduction of molecular oxygen ($O_{2}$) by the electrons, resulting in the formation of superoxide radical anions ($O_{2}^{-}$·) and hydrogen peroxide radicals (·OOH) in a rapid manner. The photo-generated holes could react with surface-adsorbed water ($H_{2}O$) or hydroxyl (${OH}^{-}$) molecules to produce hydroxyl radicals (HO·), or they could directly oxidize adsorbed dye molecules.

The formation of (HO·) radicals can be explained by the following:

$$\begin{array}{l} Ag/AuNps+light\to e_{CB}^{-}+h_{VB}^{+}\\ H_{2}O+h^{+}\to \cdot OH+H^{+}\\ O_{2}+e^{-}\to {\cdot O}_{2}^{-}\\ 2\;{\cdot O}_{2}^{-}+{2H}^{+}\to O_{2}+H_{2}O_{2}\\ H_{2}O_{2}\to 2\cdot OH\\ MB+\cdot OH\to \mathrm{Degradation}\mathrm{product} \end{array}$$

### 3.5. Reuse of the Grafted Nonwoven Membrane in the Photodegradation Process 

**Figure 8:**
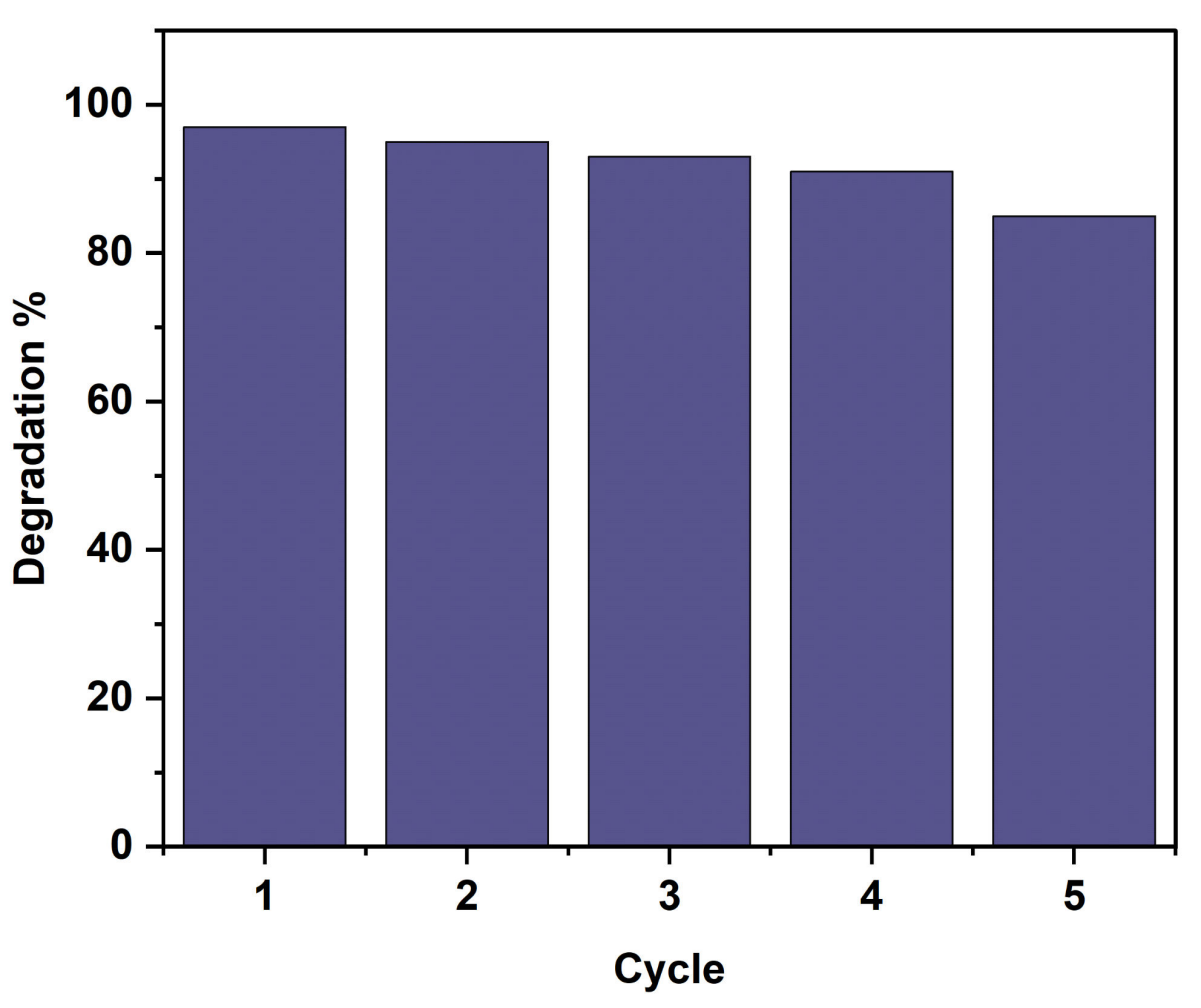
Photodegradation efficiency of the PBT/Au/Ag nonwoven membrane on the MB (1 cm^2^ in 5 mL) solution after five cycles under sunlight (90 min).

The utilization of grafted membranes offers a significant benefit in terms of the photocatalyst’s reusability. For the subsequent experiments, PBT/Au/Ag was selected to investigate its reusability under sunlight in the photodegradation of MB (5 ppm). The outcomes of the experiments are presented in Figure 8. It is evident that the PBT/Au/Ag photocatalyst can be employed multiple times, up to five cycles, with a slight reduction in photocatalytic efficiency observed over the five successive cycles. Specifically, the efficiency decreased from 97% (cycle 1) to 85% (cycle 5).

### 3.6. Antibacterial Activity of Treated Nonwoven Membrane 

**Figure 9:**
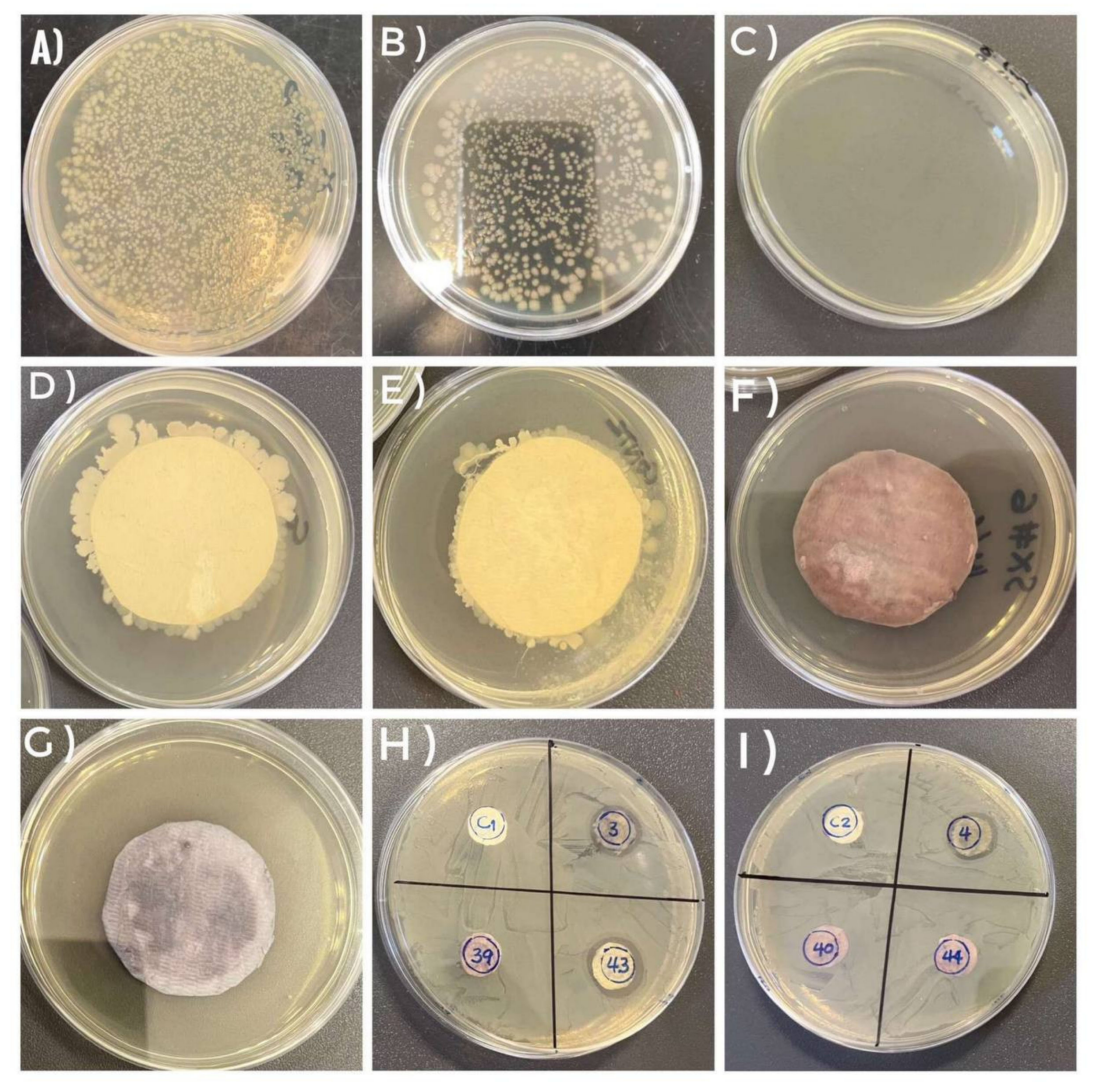
Antibacterial properties of the photo-grafted membrane. Bacterial growth of (**A**) *E. coli* suspension passed through the blank PP, (**B**) *E. coli* suspension passed through the blank PBT and (**C**) passed through PP/Au/Ag and PBT/Au/Ag (same result). (**D**,**E**) The blank PP and PBT nonwoven membrane after filtration. (**F**,**G**) PP/Au/Ag and PBT/Au/Ag membranes after filtration. (**H**,**I**) inhibition zone for different samples.

After using the nonwoven membranes to filtrate the *E. coli* suspension, the results indicate that the untreated nonwoven membrane exhibited a progressive increase in bacterial growth over time, as depicted in Figure 9A,B. While the samples modified with bimetallic nanoparticles (PP/Au/Ag and PBT/Au/Ag) both showed the same result, Figure 9C demonstrated a reduction in bacterial growth after 96 h of incubation. Subsequently, the nonwoven membrane was immediately placed onto the agar plate following to the filtration process. The conditions for the experiment were established to ensure the viability and proliferative capacity of *E. coli* on the agar plate following a 96-h incubation period. Photographs of the original PP and PBT, utilized for *E. coli* filtration, are presented in Figure 9D,E. Afterward, they were incubated on agar in a growth medium at 37 °C for 24 h. As a result, the adhesion and viability of bacteria can be observed. The fact that there are numerous colonies on the surface and around the original PP and PBT membranes proves that numerous bacteria have attached to nonwoven membrane surfaces. No single colony formed on the surface of the grafted membranes, as shown in Figure 9F,G. These findings show that the grafted nonwoven membrane does indeed have bactericidal activity, which indicates that the treated nonwoven membrane completely rendered bacteria inactive. Consequently, the filter effluent did not show any growth of bacteria, which indicates that the treated nonwoven membrane exhibits antibacterial activity.

Using an agar-gel method, the treated membrane’s antibacterial activity was also examined against *E. coli*. By calculating the zone of inhibition (ZoI) around the disk following an incubation period at 37 °C, the antibacterial properties were determined. Figure 9H,I shows the zones of inhibition for the different samples. Some samples of the treated nonwoven membrane (10 mm) showed good antibacterial activity with a zone of inhibition of 17.02 ± 0.33, 16.08 ± 0.21, and 15.94 ± 0.51 mm (Table 6). This outcome demonstrated that the bimetallic nanoparticle-treated nonwoven membrane has antibacterial properties. The untreated membrane, however, lacks any antibacterial properties.

## 4. Conclusions

In this study, we have achieved successful modification of polypropylene (PP) and poly(butylene terephthalate) (PBT) nonwoven membranes through photopolymerization using photoinitiators and nanoparticles. The surface grafting process involving polyethylene glycol diacrylate was completed with positive outcomes, leading to the highly efficient removal of methylene blue dye from water. Our findings indicate that the efficiency of dye removal is influenced by multiple factors, including the irradiation source, nonwoven membrane type, and nanoparticle composition. Notably, the PBT membrane-based samples exhibited the highest dye removal efficiency, particularly when bimetallic (Au/Ag) nanoparticles were utilized compared to monometallic (Au or Ag) counterparts. Furthermore, samples exposed to sunlight demonstrated greater degradation efficiency compared to those exposed to LED light. Among the tested samples, PBT/Au/Ag exhibited the highest percentage of dye degradation, achieving an impressive 97% removal within a short duration of 90 min under sunlight irradiation. Additionally, the modified nonwoven membranes displayed promising reusability, showing only a marginal decrease in dye removal efficiency from 97% in the first cycle to 85% in the fifth cycle. Moreover, the samples modified with bimetallic nanoparticles (PBT/Ag/Au and PP/Ag/Au) showcased notable antibacterial activity against *E. coli*.

Overall, our findings suggest that the incorporation of nanoparticles and the implementation of photo-grafting polymerization present a promising approach for enhancing the performance of polymeric membranes in water treatment applications. This study underscores the significance of surface modification in augmenting the dye removal efficiency of polymeric membranes. The ability to tailor the surface properties of these membranes through photopolymerization offers a versatile and effective means of improving their overall performance. Further research is warranted to optimize the modification process and explore its applicability in various water treatment scenarios. Nevertheless, the results obtained from this study provide valuable insights into the development of innovative and efficient methods for water treatment, with significant implications for water resource management and environmental sustainability.

## Figures and Tables

**Table 1:** The type and quantity (wt.%) of the materials were used to modify the nonwoven membrane (each formulation contains 1 wt.% of BP).

Sample	Membrane	PEGDA	${AgNO}_{3}$	${HAuCl}_{4}$
PP/Au	PP	95	-	4
PP/Ag	PP	96	3	-
PBT/Au	PBT	95	-	4
PBT/Ag	PBT	96	3	-
PP/Au/Ag	PP	95	2	2
PBT/Au/Ag	PBT	95	2	2

**Table 2:** Percentage of degradation of MB dye for 1 min and 90 min under LED with a wavelength of 419 nm irradiation.

Sample	Percentage of Degradation of MB Dye (1 min)	Percentage of Degradation of MB Dye (90 min)
PP/Au	12.5	53.8
PP/Ag	23.4	55.1
PBT/Au	12.5	56.6
PBT/Ag	22.1	62

**Table 3:** Percentage of degradation of MB dye for 1 min and 90 min under sunlight irradiation.

Sample	Percentage of Degradation of MB Dye (1 min)	Percentage of Degradation of MB Dye (90 min)
PP/Au	25	88
PP/Ag	23	85
PBT/Au	25	89
PBT/Ag	27	91

**Table 4:** Percentage of degradation of MB dyes for 1 min and 90 irradiation time under sunlight.

Sample	Percentage of Degradation of MB Dye (1 min)	Percentage of Degradation of MB Dye (90 min)
PP/Au/Ag	50	92
PBT/Au/Ag	61	97

**Table 5:** Comparison of rate constants k (min^−1^) for different irradiation sources.

Sample No.	Rate Constants k (min^−1^) LED	Rate Constants k (min^−1^) SL
PP/Au	0.00587	0.01186
PP/Ag	0.00551	0.01615
PBT/Au	0.00741	0.02018
PBT/Ag	0.0081	0.02184

**Table 6:** The values shown are zones of inhibition, given as diameter (mm). -, absence of the inhibition zone.

Sample	Zone of Inhibition (mm)
PP/Au/Ag (3)	17.02 ± 0.33
PP/Au (39)	-
PP/Ag (4)	16.08 ± 0.21
PBT/Au/Ag (43)	15.94 ± 0.51
PBT/Au (40)	-
PBT/Ag (44)	-
Control-PP (C1)	-
Control-PBT (C2)	-

## Data Availability

Not applicable.

## Supplementary Materials

The following supporting information can be downloaded at: https://www.mdpi.com/article/10.3390/polym15163378/s1, Figure S1: UV-Vis absorption spectrum of the solution; Figure S2: Calibration curve of the absorption of the AO solution (at 485 nm); Figure S3: Illustrate the D-solution method to determine tertiary amino group; Figure S4: Photograph of the samples after modification; Figure S5: SEM images of nanoparticles with their respective size; Figure S6: Kinetics of photocatalytic degradation of MB with different samples under LED light and sunlight.
