# Supplementary material for: Surface Modification of PP and PBT Nonwoven Membranes for Enhanced Efficiency in Photocatalytic MB Dye Removal and Antibacterial Activity
Source: Polymers (Basel). 2023 Aug 11;15(16):3378. doi: 10.3390/polym15163378 (PMC10459508; doi:10.3390/polym15163378)
Supplement: Supplementary file 1 [file polymers-15-03378-s001.zip › polymers-2534770-supplementary.pdf]

Surface Modification of PP and PBT Nonwoven Membranes for Enhanced Efficiency in Photocatalytic Dye Removal and Antibacterial Activity

Shahad M. Aldebasi [1], Haja Tar [1,*], Abrar S. Alnafisah [1,*], Hanène Salmi-Mani[2], Noura Kouki[1], Fahad M. Alminderej[1] and Jacques Lalevée[3]

Department of Chemistry, College of Science, Qassim University, Buraidah 51452, Saudi Arabia; 411207279@qu.edu.sa (S.M.A.); n.kouki@qu.edu.sa (N.K.); f.alminderej@qu.edu.sa (F.M.A.)

[2] Institut de Chimie Moléculaire et des Matériaux d'Orsay, Université Paris-Saclay, CNRS, 91405 Orsay cedex, France; hanene.salmi@u-psud.fr

[3] CNRS, IS2M UMR 7361, Université de Haute-Alsace, F-68100 Mulhouse, France; jacques.lalevee@uha.fr

* Correspondence: h.tar@qu.edu.sa (H.T.); alnafisaha@qu.edu.sa (A.S.A.); Tel.: +966-163013490 (H.T.)

1.Tertiary amino group determination on the surface of PBT and PP

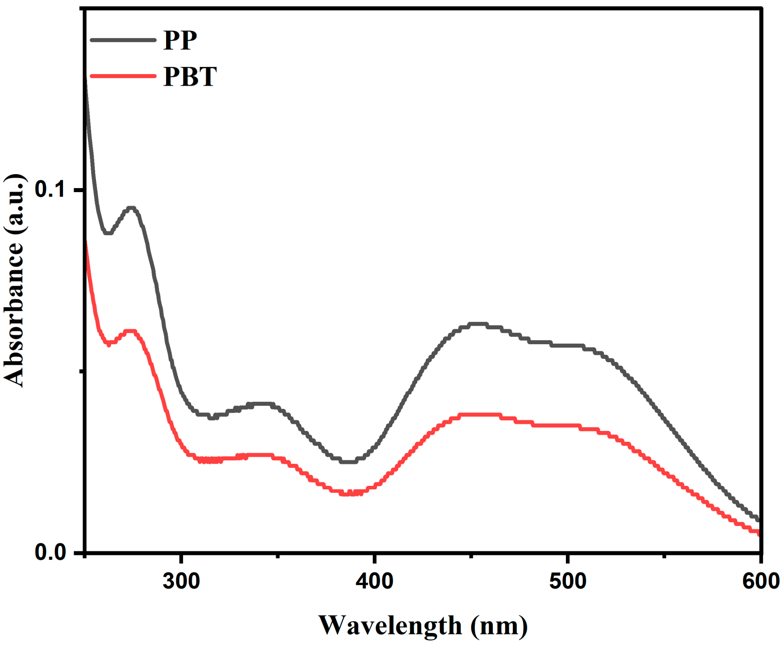

Figure S1. UV-Vis absorption spectrum of the solution.

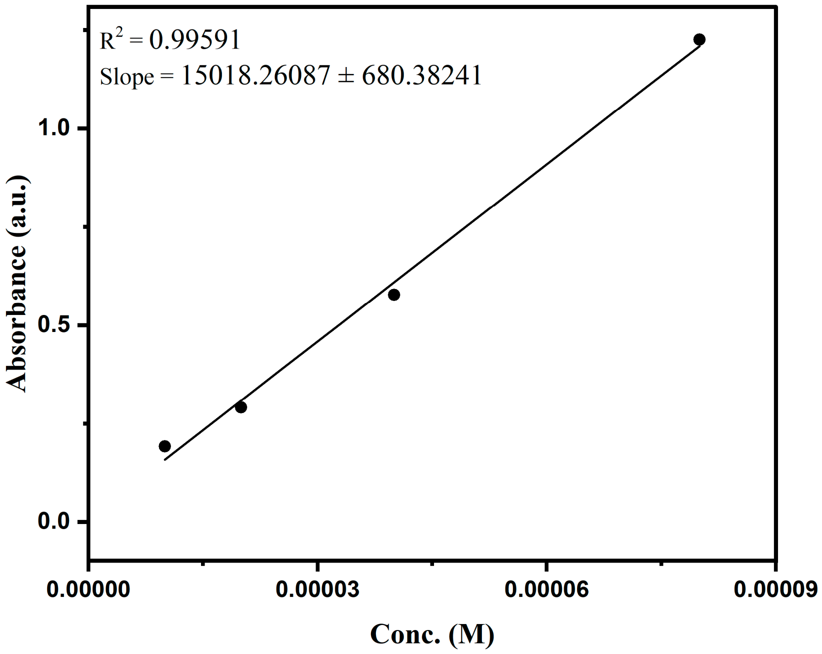

Figure S2. Calibration curve of the absorption of the AO solution (at 485 nm).

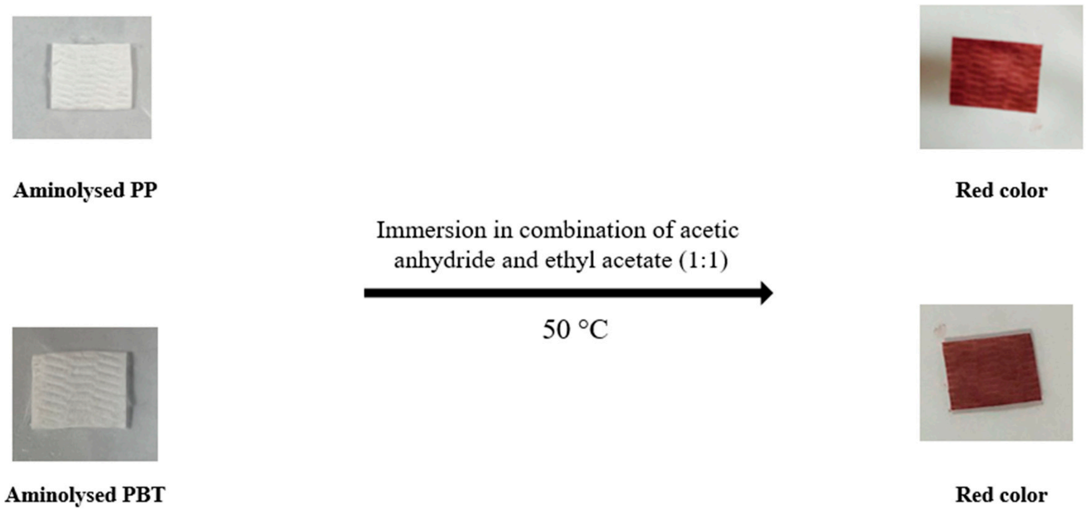

Figure S3. Illustrate the D-solution method to determine tertiary amino group.

2. PP and PBT after grafting

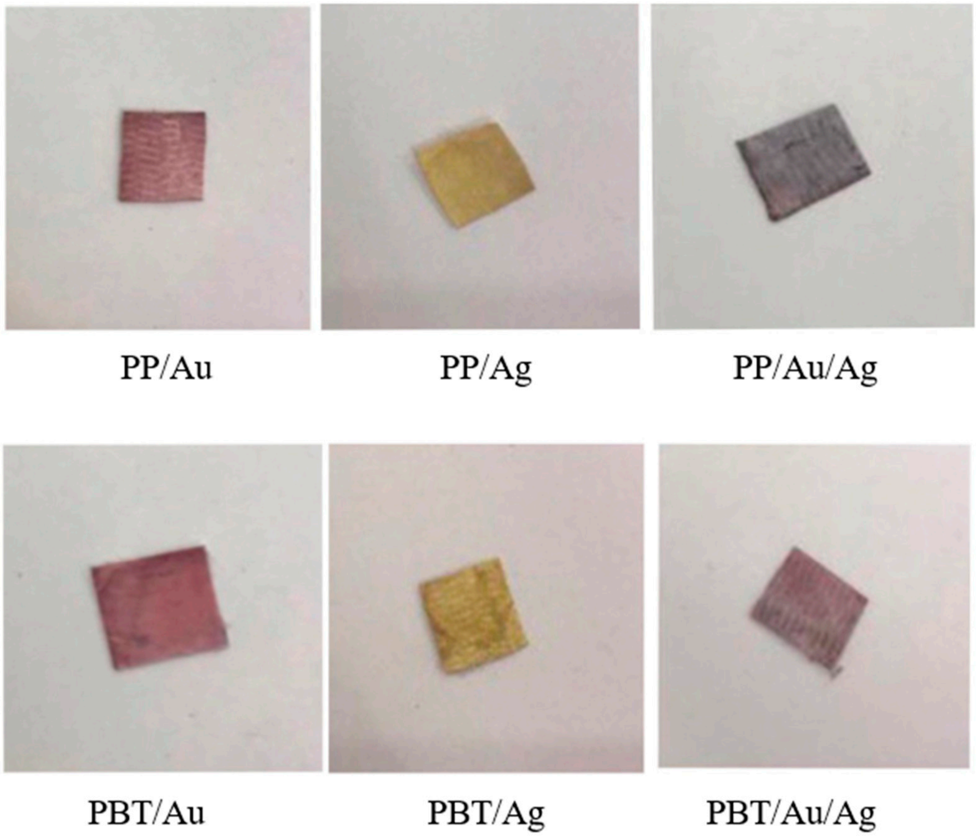

Figure S4. Photograph of the samples after modification.

3. SEM characterization

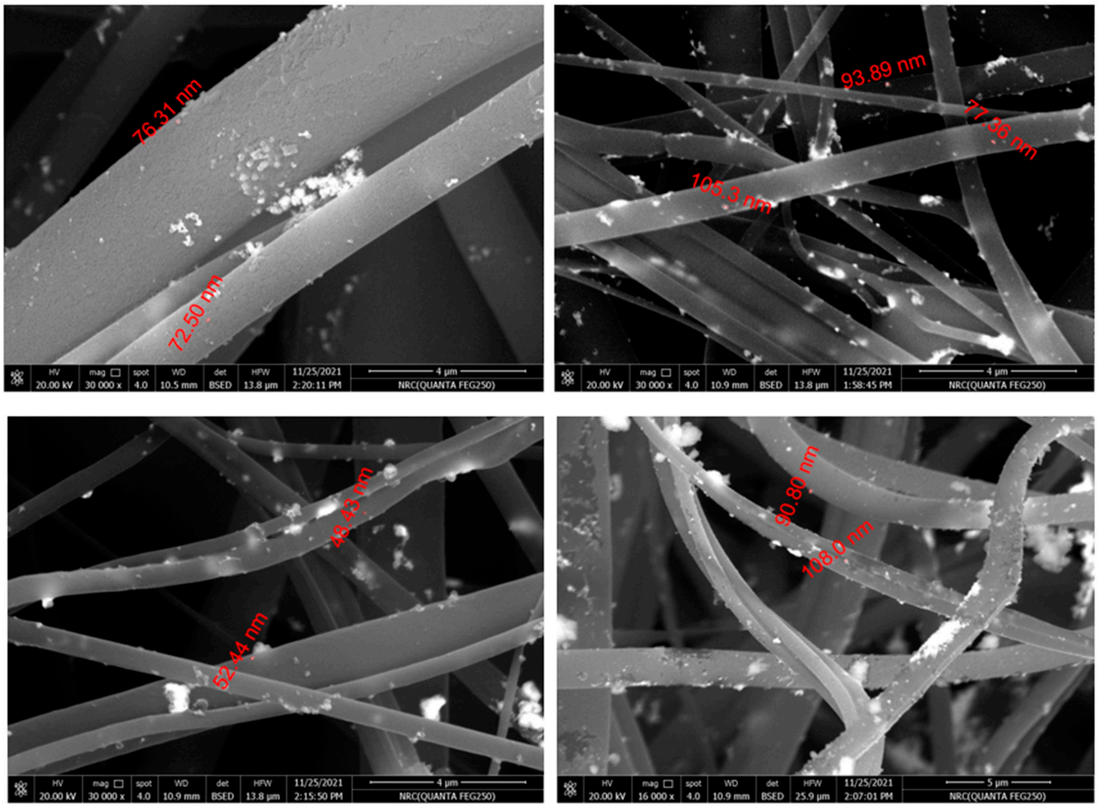

Figure S5. SEM images of nanoparticles with their respective size.

4. Kinetics study

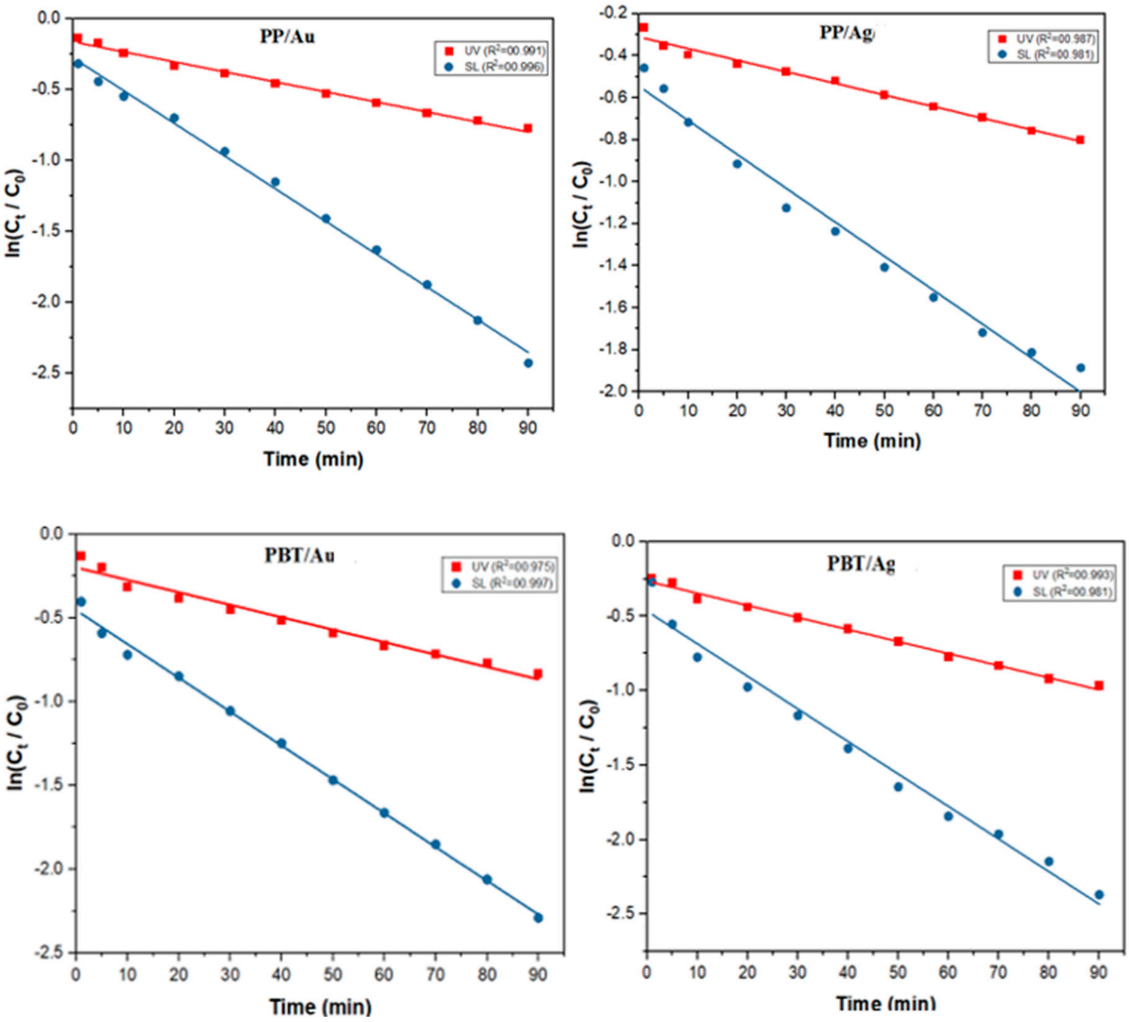

Figure S6. Kinetics of photocatalytic degradation of MB with different samples under LED light and sunlight.
